# 4-Hydr­oxy-*N*′-(3,5-dichloro-2-hydroxy­benzyl­idene)benzohydrazide

**DOI:** 10.1107/S1600536809020820

**Published:** 2009-06-06

**Authors:** Chong-Gui Ren

**Affiliations:** a Department of Chemistry and Chemical Engineering, Zaozhuang University, Zaozhuang Shandong 277160, People’s Republic of China

## Abstract

In the title compound, C_14_H_10_Cl_2_N_2_O_3_, the dihedral angle between the two benzene rings is 5.1 (2)°. The mol­ecule adopts an *E* configuration with respect to the C=N bond and an intra­molecular O—H⋯N inter­action is present. In the crystal structure, mol­ecules are linked through inter­molecular N—H⋯O and O—H⋯O hydrogen bonds.

## Related literature

For the biological properties of Schiff base compounds, see: Jeewoth *et al.* (1999 ▶); Ren *et al.* (2002 ▶); Eltayeb *et al.* (2008 ▶); Sinha *et al.* (2008 ▶). For metal complexes of Schiff base compounds, see: Shivakumar *et al.* (2008 ▶); Prabhakaran *et al.* (2006 ▶); Dhar *et al.* (2005 ▶). For related structures, see: Cui *et al.* (2007 ▶); Jing *et al.* (2007 ▶); Ma *et al.* (2008 ▶); Salhin *et al.* (2007 ▶); Lin *et al.* (2007 ▶); Alhadi *et al.* (2008 ▶); Xue *et al.* (2008 ▶); Wang *et al.* (2008 ▶); Lu (2008 ▶); Diao *et al.* (2008 ▶); Qiu (2009 ▶); Mohd Lair *et al.* (2009*a* ▶,*b* ▶). For reference structural data, see: Allen *et al.* (1987 ▶).
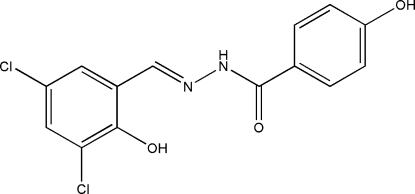


## Experimental

### 

#### Crystal data

C_14_H_10_Cl_2_N_2_O_3_
*M* *_r_* = 325.14
Monoclinic, 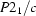

*a* = 8.030 (1) Å
*b* = 13.546 (2) Å
*c* = 13.433 (2) Å
β = 107.247 (2)°
*V* = 1395.5 (3) Å^3^
*Z* = 4
Mo *K*α radiation
μ = 0.48 mm^−1^
*T* = 298 K
0.20 × 0.20 × 0.18 mm

#### Data collection

Bruker SMART CCD area-detector diffractometer
Absorption correction: multi-scan (*SADABS*; Sheldrick, 1996 ▶) *T*_min_ = 0.911, *T*_max_ = 0.919
8463 measured reflections
3039 independent reflections
2324 reflections with *I* > 2σ(*I*)
*R*_int_ = 0.023

#### Refinement

*R*[*F* ^2^ > 2σ(*F* ^2^)] = 0.039
*wR*(*F* ^2^) = 0.107
*S* = 1.03
3039 reflections
195 parameters
1 restraint
H atoms treated by a mixture of independent and constrained refinement
Δρ_max_ = 0.22 e Å^−3^
Δρ_min_ = −0.36 e Å^−3^

### 

Data collection: *SMART* (Bruker, 2002 ▶); cell refinement: *SAINT* (Bruker, 2002 ▶); data reduction: *SAINT*; program(s) used to solve structure: *SHELXS97* (Sheldrick, 2008 ▶); program(s) used to refine structure: *SHELXL97* (Sheldrick, 2008 ▶); molecular graphics: *SHELXTL* (Sheldrick, 2008 ▶); software used to prepare material for publication: *SHELXL97*.

## Supplementary Material

Crystal structure: contains datablocks global, I. DOI: 10.1107/S1600536809020820/bx2214sup1.cif

Structure factors: contains datablocks I. DOI: 10.1107/S1600536809020820/bx2214Isup2.hkl

Additional supplementary materials:  crystallographic information; 3D view; checkCIF report

## Figures and Tables

**Table 1:** Hydrogen-bond geometry (Å, °)

*D*—H⋯*A*	*D*—H	H⋯*A*	*D*⋯*A*	*D*—H⋯*A*
O1—H1⋯N1	0.82	1.90	2.6164 (19)	145
O3—H3⋯O2^i^	0.82	1.85	2.658 (2)	168
N2—H2⋯O3^ii^	0.899 (10)	2.204 (16)	3.000 (2)	147 (2)

Symmetry codes: (i) 
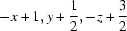
; (ii) 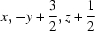.

**Fig. 1.**
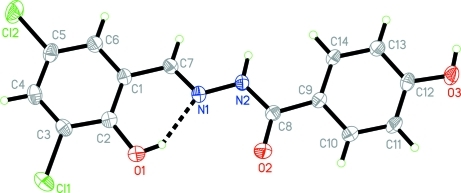
The molecular structure of the compound with 30% probability ellipsoids. The intramolecular O–H···N hydrogen bond is shown as a dashed line.

**Fig. 2.**
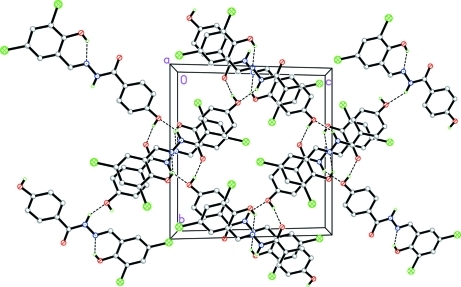
Molecular packing of the compound with hydrogen bonds drawn as dashed lines. Hydrogen atoms not involved in hydrogen bonding have been omitted.

## supplementary crystallographic information

### Comment

The Schiff base compounds show excellent biological properties (Jeewoth *et al.*, 1999; Ren *et al.*, 2002; Eltayeb *et al.*, 2008; Sinha *et al.*, 2008). Moreover, the Schiff base compounds have been widely used as versatile ligands in coordination chemistry (Shivakumar *et al.*, 2008; Prabhakaran *et al.*, 2006; Dhar *et al.*, 2005). We report here the crystal structure of the title compound. In the title compound, Fig. 1, the dihedral angle between the two benzene rings is 5.1 (2)°. All the bond lengths are within normal values (Allen *et al.*, 1987) and comparable to those in other similar compounds (Cui *et al.*, 2007; Jing *et al.*, 2007; Ma *et al.*, 2008; Salhin *et al.*, 2007; Lin *et al.*, 2007; Alhadi *et al.*, 2008; Xue *et al.*, 2008; Wang *et al.*, 2008; Lu, 2008; Diao *et al.*, 2008; Qiu, 2009; Mohd Lair *et al.*, 2009*a*,b). —H···N OIn the crystal structure, molecules are linked through intermolecular N–H···O and O–H···O hydrogen bonds (Table 1), Fig. 2.

### Experimental

All the starting materials were obtained with AR grade from Lancaster. 3,5-Dichloro-2-hydroxybenzaldehyde (1.0 mmol, 192.2 mg) and 4-hydroxybenzohydrazide (1.0 mmol, 152.2 mg) were refluxed in  30 ml methanol solution for 30 min  giving a clear yellow solution. Yellow block-shaped single crystals of the compound were obtained by slow evaporation of the solution for a week at room temperature.

### Refinement

H2 was located from a difference Fourier map and refined isotropically, with the N–H distance restrained to 0.90 (1) Å, and with *U*_iso_ restrained to 0.08 Å^2^. Other H atoms were constrained to ideal geometries, with d(C–H) = 0.93 Å, d(O–H) = 0.82 Å, and with *U*_iso_(H) = 1.2*U*_eq_(C) and 1.5*U*_eq_(O).

### Crystal data

**Table:**

C_14_H_10_Cl_2_N_2_O_3_	*F*(000) = 664
*M**_r_* = 325.14	*D*_x_ = 1.547 Mg m^−^^3^
Monoclinic, *P*2_1_/*c*	Mo *K*α radiation, λ = 0.71073 Å
Hall symbol: -P 2ybc	Cell parameters from 2634 reflections
*a* = 8.030 (1) Å	θ = 2.2–26.0°
*b* = 13.546 (2) Å	µ = 0.48 mm^−^^1^
*c* = 13.433 (2) Å	*T* = 298 K
β = 107.247 (2)°	Block, yellow
*V* = 1395.5 (3) Å^3^	0.20 × 0.20 × 0.18 mm
*Z* = 4	

### Data collection

**Table:**

Bruker SMART CCD area-detector diffractometer	3039 independent reflections
Radiation source: fine-focus sealed tube	2324 reflections with *I* > 2σ(*I*)
graphite	*R*_int_ = 0.023
ω scans	θ_max_ = 27.0°, θ_min_ = 2.2°
Absorption correction: multi-scan (*SADABS*; Sheldrick, 1996)	*h* = −10→9
*T*_min_ = 0.911, *T*_max_ = 0.919	*k* = −17→17
8463 measured reflections	*l* = −17→14

### Refinement

**Table:**

Refinement on *F*^2^	Primary atom site location: structure-invariant direct methods
Least-squares matrix: full	Secondary atom site location: difference Fourier map
*R*[*F*^2^ > 2σ(*F*^2^)] = 0.039	Hydrogen site location: inferred from neighbouring sites
*wR*(*F*^2^) = 0.107	H atoms treated by a mixture of independent and constrained refinement
*S* = 1.03	*w* = 1/[σ^2^(*F*_o_^2^) + (0.0503*P*)^2^ + 0.3954*P*] where *P* = (*F*_o_^2^ + 2*F*_c_^2^)/3
3039 reflections	(Δ/σ)_max_ = 0.001
195 parameters	Δρ_max_ = 0.22 e Å^−^^3^
1 restraint	Δρ_min_ = −0.36 e Å^−^^3^

### Special details

**Table:**

Geometry. All e.s.d.'s (except the e.s.d. in the dihedral angle between two l.s. planes) are estimated using the full covariance matrix. The cell e.s.d.'s are taken into account individually in the estimation of e.s.d.'s in distances, angles and torsion angles; correlations between e.s.d.'s in cell parameters are only used when they are defined by crystal symmetry. An approximate (isotropic) treatment of cell e.s.d.'s is used for estimating e.s.d.'s involving l.s. planes.
Refinement. Refinement of *F*^2^ against ALL reflections. The weighted *R*-factor *wR* and goodness of fit *S* are based on *F*^2^, conventional *R*-factors *R* are based on *F*, with *F* set to zero for negative *F*^2^. The threshold expression of *F*^2^ > σ(*F*^2^) is used only for calculating *R*-factors(gt) *etc*. and is not relevant to the choice of reflections for refinement. *R*-factors based on *F*^2^ are statistically about twice as large as those based on *F*, and *R*- factors based on ALL data will be even larger.

### Fractional atomic coordinates and isotropic or equivalent isotropic displacement parameters (Å^2^)

**Table:**

	*x*	*y*	*z*	*U*_iso_*/*U*_eq_	
Cl1	−0.05205 (8)	0.19042 (4)	1.13049 (5)	0.0682 (2)	
Cl2	0.17799 (11)	0.43904 (5)	1.45283 (5)	0.0843 (2)	
N1	0.25047 (18)	0.49746 (11)	1.00303 (11)	0.0382 (3)	
N2	0.31078 (19)	0.56552 (10)	0.94624 (11)	0.0387 (3)	
O1	0.08301 (19)	0.33479 (10)	1.01678 (10)	0.0512 (3)	
H1	0.1314	0.3745	0.9885	0.077*	
O2	0.26379 (19)	0.45814 (10)	0.81320 (10)	0.0540 (4)	
O3	0.55673 (19)	0.80001 (10)	0.59831 (10)	0.0512 (3)	
H3	0.6200	0.8433	0.6322	0.077*	
C1	0.1834 (2)	0.45241 (12)	1.15726 (13)	0.0369 (4)	
C2	0.1046 (2)	0.36288 (13)	1.11609 (14)	0.0384 (4)	
C3	0.0458 (2)	0.30054 (13)	1.18096 (15)	0.0422 (4)	
C4	0.0667 (2)	0.32330 (14)	1.28362 (15)	0.0467 (5)	
H4	0.0275	0.2804	1.3259	0.056*	
C5	0.1470 (3)	0.41115 (15)	1.32275 (14)	0.0479 (5)	
C6	0.2037 (2)	0.47558 (14)	1.26095 (14)	0.0436 (4)	
H6	0.2558	0.5348	1.2885	0.052*	
C7	0.2478 (2)	0.52136 (13)	1.09426 (13)	0.0388 (4)	
H7	0.2873	0.5833	1.1208	0.047*	
C8	0.3119 (2)	0.54031 (13)	0.84892 (13)	0.0366 (4)	
C9	0.3736 (2)	0.61545 (12)	0.78824 (13)	0.0354 (4)	
C10	0.3425 (3)	0.59603 (15)	0.68236 (14)	0.0468 (4)	
H10	0.2799	0.5400	0.6532	0.056*	
C11	0.4035 (3)	0.65890 (16)	0.62059 (14)	0.0497 (5)	
H11	0.3815	0.6454	0.5500	0.060*	
C12	0.4974 (2)	0.74196 (13)	0.66340 (13)	0.0402 (4)	
C13	0.5276 (2)	0.76339 (13)	0.76825 (13)	0.0404 (4)	
H13	0.5893	0.8199	0.7969	0.049*	
C14	0.4656 (2)	0.70024 (13)	0.82994 (13)	0.0383 (4)	
H14	0.4856	0.7147	0.9002	0.046*	
H2	0.352 (3)	0.6232 (11)	0.9766 (18)	0.080*	

### Atomic displacement parameters (Å^2^)

**Table:**

	*U*^11^	*U*^22^	*U*^33^	*U*^12^	*U*^13^	*U*^23^
Cl1	0.0753 (4)	0.0500 (3)	0.0806 (4)	−0.0224 (3)	0.0252 (3)	−0.0019 (3)
Cl2	0.1359 (6)	0.0822 (5)	0.0491 (3)	−0.0081 (4)	0.0492 (4)	−0.0052 (3)
N1	0.0398 (8)	0.0373 (7)	0.0391 (8)	0.0010 (6)	0.0143 (6)	0.0045 (6)
N2	0.0462 (8)	0.0364 (8)	0.0361 (8)	−0.0036 (6)	0.0165 (7)	0.0022 (6)
O1	0.0629 (9)	0.0480 (8)	0.0433 (7)	−0.0110 (6)	0.0165 (6)	−0.0057 (6)
O2	0.0730 (10)	0.0428 (7)	0.0462 (8)	−0.0120 (7)	0.0174 (7)	−0.0068 (6)
O3	0.0679 (10)	0.0503 (8)	0.0392 (7)	0.0019 (6)	0.0218 (7)	0.0104 (6)
C1	0.0357 (9)	0.0358 (9)	0.0422 (9)	0.0033 (7)	0.0160 (7)	0.0027 (7)
C2	0.0340 (9)	0.0394 (9)	0.0421 (9)	0.0042 (7)	0.0117 (7)	0.0021 (7)
C3	0.0347 (9)	0.0383 (9)	0.0543 (11)	0.0006 (7)	0.0140 (8)	0.0040 (8)
C4	0.0438 (10)	0.0478 (11)	0.0553 (11)	0.0066 (8)	0.0249 (9)	0.0130 (9)
C5	0.0563 (12)	0.0525 (11)	0.0413 (10)	0.0068 (9)	0.0241 (9)	0.0032 (8)
C6	0.0500 (11)	0.0404 (9)	0.0452 (10)	0.0000 (8)	0.0212 (9)	−0.0030 (8)
C7	0.0399 (9)	0.0355 (9)	0.0427 (10)	−0.0012 (7)	0.0147 (8)	−0.0003 (7)
C8	0.0359 (9)	0.0382 (9)	0.0343 (9)	0.0031 (7)	0.0080 (7)	0.0008 (7)
C9	0.0345 (9)	0.0392 (9)	0.0326 (8)	0.0059 (7)	0.0102 (7)	0.0010 (7)
C10	0.0527 (11)	0.0510 (11)	0.0347 (9)	−0.0067 (9)	0.0098 (8)	−0.0043 (8)
C11	0.0588 (12)	0.0615 (12)	0.0276 (9)	−0.0009 (10)	0.0107 (8)	−0.0009 (8)
C12	0.0453 (10)	0.0419 (10)	0.0351 (9)	0.0116 (8)	0.0144 (7)	0.0106 (7)
C13	0.0468 (10)	0.0376 (9)	0.0373 (9)	0.0016 (8)	0.0132 (8)	0.0003 (7)
C14	0.0453 (10)	0.0412 (9)	0.0299 (8)	0.0037 (7)	0.0135 (7)	−0.0014 (7)

### Geometric parameters (Å, °)

**Table:**

Cl1—C3	1.7281 (19)	C4—C5	1.381 (3)
Cl2—C5	1.7321 (19)	C4—H4	0.9300
N1—C7	1.274 (2)	C5—C6	1.372 (3)
N1—N2	1.3732 (19)	C6—H6	0.9300
N2—C8	1.354 (2)	C7—H7	0.9300
N2—H2	0.899 (10)	C8—C9	1.478 (2)
O1—C2	1.348 (2)	C9—C14	1.390 (2)
O1—H1	0.8200	C9—C10	1.394 (2)
O2—C8	1.228 (2)	C10—C11	1.376 (3)
O3—C12	1.363 (2)	C10—H10	0.9300
O3—H3	0.8200	C11—C12	1.381 (3)
C1—C6	1.389 (2)	C11—H11	0.9300
C1—C2	1.403 (2)	C12—C13	1.387 (2)
C1—C7	1.455 (2)	C13—C14	1.383 (2)
C2—C3	1.393 (2)	C13—H13	0.9300
C3—C4	1.374 (3)	C14—H14	0.9300
			
C7—N1—N2	118.23 (15)	N1—C7—C1	120.45 (16)
C8—N2—N1	118.12 (14)	N1—C7—H7	119.8
C8—N2—H2	123.3 (16)	C1—C7—H7	119.8
N1—N2—H2	118.5 (16)	O2—C8—N2	120.98 (16)
C2—O1—H1	109.5	O2—C8—C9	121.77 (15)
C12—O3—H3	109.5	N2—C8—C9	117.26 (15)
C6—C1—C2	119.72 (16)	C14—C9—C10	118.68 (16)
C6—C1—C7	118.80 (16)	C14—C9—C8	124.85 (15)
C2—C1—C7	121.47 (15)	C10—C9—C8	116.40 (16)
O1—C2—C3	118.68 (16)	C11—C10—C9	120.67 (18)
O1—C2—C1	123.01 (16)	C11—C10—H10	119.7
C3—C2—C1	118.30 (16)	C9—C10—H10	119.7
C4—C3—C2	121.93 (17)	C10—C11—C12	120.06 (16)
C4—C3—Cl1	119.57 (14)	C10—C11—H11	120.0
C2—C3—Cl1	118.49 (15)	C12—C11—H11	120.0
C3—C4—C5	118.66 (17)	O3—C12—C11	117.03 (15)
C3—C4—H4	120.7	O3—C12—C13	122.78 (17)
C5—C4—H4	120.7	C11—C12—C13	120.19 (16)
C6—C5—C4	121.27 (17)	C14—C13—C12	119.56 (17)
C6—C5—Cl2	119.84 (16)	C14—C13—H13	120.2
C4—C5—Cl2	118.89 (15)	C12—C13—H13	120.2
C5—C6—C1	120.10 (17)	C13—C14—C9	120.82 (15)
C5—C6—H6	120.0	C13—C14—H14	119.6
C1—C6—H6	120.0	C9—C14—H14	119.6
			
C7—N1—N2—C8	−179.36 (15)	C6—C1—C7—N1	171.54 (16)
C6—C1—C2—O1	−178.94 (16)	C2—C1—C7—N1	−7.3 (3)
C7—C1—C2—O1	−0.1 (3)	N1—N2—C8—O2	−1.5 (2)
C6—C1—C2—C3	1.3 (2)	N1—N2—C8—C9	178.67 (14)
C7—C1—C2—C3	−179.91 (16)	O2—C8—C9—C14	−164.75 (17)
O1—C2—C3—C4	178.59 (17)	N2—C8—C9—C14	15.1 (2)
C1—C2—C3—C4	−1.6 (3)	O2—C8—C9—C10	12.3 (2)
O1—C2—C3—Cl1	0.0 (2)	N2—C8—C9—C10	−167.92 (16)
C1—C2—C3—Cl1	179.75 (13)	C14—C9—C10—C11	0.8 (3)
C2—C3—C4—C5	0.6 (3)	C8—C9—C10—C11	−176.42 (17)
Cl1—C3—C4—C5	179.25 (14)	C9—C10—C11—C12	0.3 (3)
C3—C4—C5—C6	0.7 (3)	C10—C11—C12—O3	178.78 (17)
C3—C4—C5—Cl2	−178.61 (15)	C10—C11—C12—C13	−1.3 (3)
C4—C5—C6—C1	−1.0 (3)	O3—C12—C13—C14	−179.03 (16)
Cl2—C5—C6—C1	178.29 (14)	C11—C12—C13—C14	1.0 (3)
C2—C1—C6—C5	0.0 (3)	C12—C13—C14—C9	0.1 (3)
C7—C1—C6—C5	−178.85 (16)	C10—C9—C14—C13	−1.0 (3)
N2—N1—C7—C1	179.32 (14)	C8—C9—C14—C13	175.92 (16)

### Hydrogen-bond geometry (Å, °)

**Table:**

*D*—H···*A*	*D*—H	H···*A*	*D*···*A*	*D*—H···*A*
O1—H1···N1	0.82	1.90	2.6164 (19)	145
O3—H3···O2^i^	0.82	1.85	2.658 (2)	168
N2—H2···O3^ii^	0.90 (1)	2.20 (2)	3.000 (2)	147 (2)

Symmetry codes: (i) −*x*+1, *y*+1/2, −*z*+3/2; (ii) *x*, −*y*+3/2, *z*+1/2.
